# Specific activation of pro-Infliximab enhances selectivity and safety of rheumatoid arthritis therapy

**DOI:** 10.1371/journal.pbio.3000286

**Published:** 2019-06-13

**Authors:** Yun-Chi Lu, Chih-Hung Chuang, Kuo-Hsiang Chuang, I-Ju Chen, Bo-Cheng Huang, Wen-Han Lee, Hsin-Ell Wang, Jia-Je Li, Yi-An Cheng, Kai-Wen Cheng, Jaw-Yuan Wang, Yuan-Chin Hsieh, Wen-Wei Lin, Tian-Lu Cheng

**Affiliations:** 1 Graduate Institute of Medicine, Kaohsiung Medical University, Kaohsiung, Taiwan; 2 Department of Medical Laboratory Science and Biotechnology, College of Health Sciences, Kaohsiung Medical University, Kaohsiung, Taiwan; 3 Drug Development and Value Creation Research Center, Kaohsiung Medical University, Kaohsiung, Taiwan; 4 Graduate Institute of Pharmacognosy, Taipei Medical University, Taipei, Taiwan; 5 Department of Biomedical Science and Environmental Biology, Kaohsiung Medical University, Kaohsiung, Taiwan; 6 Institute of Biomedical Sciences, National Sun Yat-Sen University, Kaohsiung, Taiwan; 7 Department of Biomedical Imaging and Radiological Sciences, National Yang-Ming University, Taipei, Taiwan; 8 Biophotonics and Molecular Imaging Research Center, National Yang-Ming University, Taipei, Taiwan; 9 Graduate Institute of Clinical Medicine, College of Medicine, Kaohsiung Medical University, Kaohsiung, Taiwan; 10 Division of Gastroenterology and General Surgery, Department of Surgery, Kaohsiung Medical University Hospital, Kaohsiung Medical University, Kaohsiung, Taiwan; 11 Department of Laboratory Medicine, School of Medicine, College of Medicine, Kaohsiung Medical University, Kaohsiung, Taiwan; 12 Department of Medical Research, Kaohsiung Medical University Hospital, Kaohsiung, Taiwan; New York University School of Medicine, UNITED STATES

## Abstract

During rheumatoid arthritis (RA) treatment, long-term injection of antitumor necrosis factor α antibodies (anti-TNFα Abs) may induce on-target toxicities, including severe infections (tuberculosis [TB] or septic arthritis) and malignancy. Here, we used an immunoglobulin G1 (IgG1) hinge as an Ab lock to cover the TNFα-binding site of Infliximab by linking it with matrix metalloproteinase (MMP) -2/9 substrate to generate pro-Infliximab that can be specifically activated in the RA region to enhance the selectivity and safety of treatment. The Ab lock significantly inhibits the TNFα binding and reduces the anti-idiotypic (anti-Id) Ab binding to pro-Infliximab by 395-fold, 108-fold compared with Infliximab, respectively, and MMP-2/9 can completely restore the TNFα neutralizing ability of pro-Infliximab to block TNFα downstream signaling. Pro-Infliximab was only selectively activated in the disease site (mouse paws) and presented similar pharmacokinetics (PKs) and bio-distribution to Infliximab. Furthermore, pro-Infliximab not only provided equivalent therapeutic efficacy to Infliximab but also maintained mouse immunity against *Listeria* infection in the RA mouse model, leading to a significantly higher survival rate (71%) than that of the Infliximab treatment group (0%). The high-selectivity pro-Infliximab maintains host immunity and keeps the original therapeutic efficiency, providing a novel strategy for RA therapy.

## Abstract 2

During treatment of rheumatoid arthritis, systemic administration of anti-TNFα antibodies may induce on-target toxicities, limiting their application. The incorporation of IgG1 hinge as an antibody lock generates a pro-Infliximab whose activation is specific to the disease region, enabling safer RA therapy.

## Introduction

Antitumor necrosis factor α antibodies (anti-TNFα Abs) constitute a major advance in rheumatoid arthritis (RA) therapy in the clinic, as targeting TNFα in the disease region can reduce pathological inflammation and efficiently inhibit RA progression [1]. In addition to their biological importance, anti-TNF biologics also constitute the most profitable drug class in history, exceeding US$25 billion total sales globally [2]. However, TNFα is not only a critical proinflammation factor for RA patients but also a key immune modulator of host defense and tumor growth control [3]. The United States Food and Drug Administration (FDA) has reported that systemic injection of anti-TNFα Abs (Infliximab [4] and Adalimumab [5]) can lead to serious adverse effects (on-target toxicities) such as severe infections [6, 7], reactivation of viral infections (hepatitis or herpes zoster) [8, 9], and elevated risk of malignancy [4, 5, 10]. Singh and colleagues indicated the risk of serious infections is increased 30% during the treatment of anti-TNFα therapy [11], and common infections such as tuberculosis (TB), bacterial sepsis, *Streptococcus pneumoniae*, and *Listeria monocytogenes* might lead to hospitalization or death. In addition, Viganò and colleagues reported the reactivation of hepatitis B (HBV), leading to severe rheumatic complications in 23 HBV surface antigen (HBsAg)-positive patients treated with TNFα inhibitors, leading to nine cases of fulminant hepatitis, four deaths, and one liver transplantation [9]. Moreover, Mariette and colleagues suggested that the anti-TNFα Abs increase the risk of lymphoma 2- to 3-fold compared with the general population [12]. Thus, there is an urgent need to solve the on-target toxicities that accompany anti-TNFα therapy to provide higher selectivity and a safe treatment for RA patients.

Several strategies have been used in the clinic to prevent the on-target toxicities, which are induced by anti-TNFα therapies. For example, vaccination can prevent the risk of pneumonia and HBV; however, not all infections can be prevented by vaccine (e.g., listeriosis) [13]. Further, the systemic immunosuppression of anti-TNFα therapies hampers the immunogenicity of these vaccines, and in addition, live vaccine (i.e., herpes zoster) was contraindicated in anti-TNFα-treated patients, as it may cause infection from the vaccine virus strain. Therefore, immunosuppressant medication limits the application of vaccinations in RA patients. In addition, screening for latent TB, HBV, and cancer before initiation of anti-TNFα therapies has been recommended to perform safer treatment. Nevertheless, 10% of RA patients still suffer from new TB infection [14]; 12.5% of inactive HBsAg carriers with arthritis showed HBV reactivation due to low or undetectable HBV [15]. Systemic immunosuppression associated with anti-TNFα therapies is widely believed to be a confounding factor in infection and HBV reactivation development. On the other hand, to prevent the risk and recurrence of lymphoma associated with anti-TNFα Abs for RA patients, rituximab (anti-CD20 Ab, Mabthera) was recommended to replace anti-TNFα Abs, which can be a dual agent that inhibits the progression of lymphoma and RA disease activity [16]. However, the systemic injection of rituximab also caused on-target toxicities, including fatal infusion and progressive multifocal leukoencephalopathy [17]. Therefore, it is desirable to develop an anti-TNFα Ab that is highly selective to the RA region to enhance safety for use in RA.

**Fig 1:**
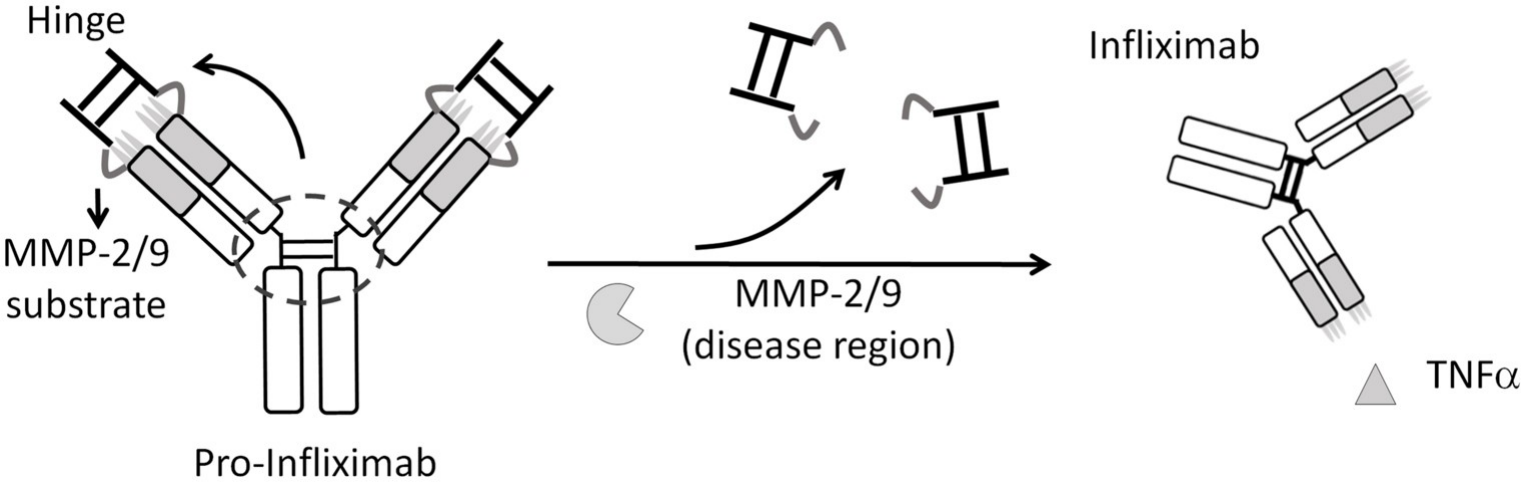
An autologous hinge as an Ab lock to enhance the selectivity and maintain the host immunity of Infliximab. We used an autologous IgG1 hinge as Ab lock to cover the antigen-binding site of Infliximab by using MMP-2/9 substrate linker to generate pro-Infliximab. Upon protease activation at the RA region, the Ab lock was released, and the pro-Infliximab could specifically activate and neutralize TNFα at the disease site to inhibit RA progression. Ab, antibody; IgG1, immunoglobulin G1; MMP, matrix metalloproteinase; RA, rheumatoid arthritis; TNFα, tumor necrosis factor α.

In this study, we developed an “antibody lock” (Ab lock) that provides Infliximab with disease selectivity. We used the autologous hinge region as an Ab lock to block the antigen-binding site of Infliximab through matrix metalloproteinase (MMP) -2/9 substrate as a linker to generate pro-Infliximab. After the Ab lock is removed by expression of MMP-2/9 in the disease region, the cleaved pro-Infliximab is expected to be specifically activated and neutralize the target antigen to suppress RA progression (Fig 1). We first examined the masking efficiency of the Ab lock on pro-Infliximab and then confirmed whether MMP-2/9 treatment could completely restore the binding ability of the pro-Infliximab to block the TNFα downstream signaling. Next, we tested whether the Ab lock could prevent the binding of anti-idiotypic (anti-Id) Ab to pro-Infliximab by immunoassay. We compared the biodistribution, serum half-life, MMP-2/9–dependent selective activation, and therapeutic efficacy of pro-Infliximab with Infliximab in mice. Finally, we tested whether pro-Infliximab reduces the risk of opportunistic infection and extended adverse events in an RA mouse model challenged with Listeria, using Infliximab as a control. The successful development of a highly selective pro-Infliximab potentially provides a safer option to prevent the systemic on-target toxicities associated with anti-TNFα therapies and improve the quality of life of patients.

## Results

### Ab lock can reversibly mask the TNFα-neutralizing ability of Infliximab

**Fig 2:**
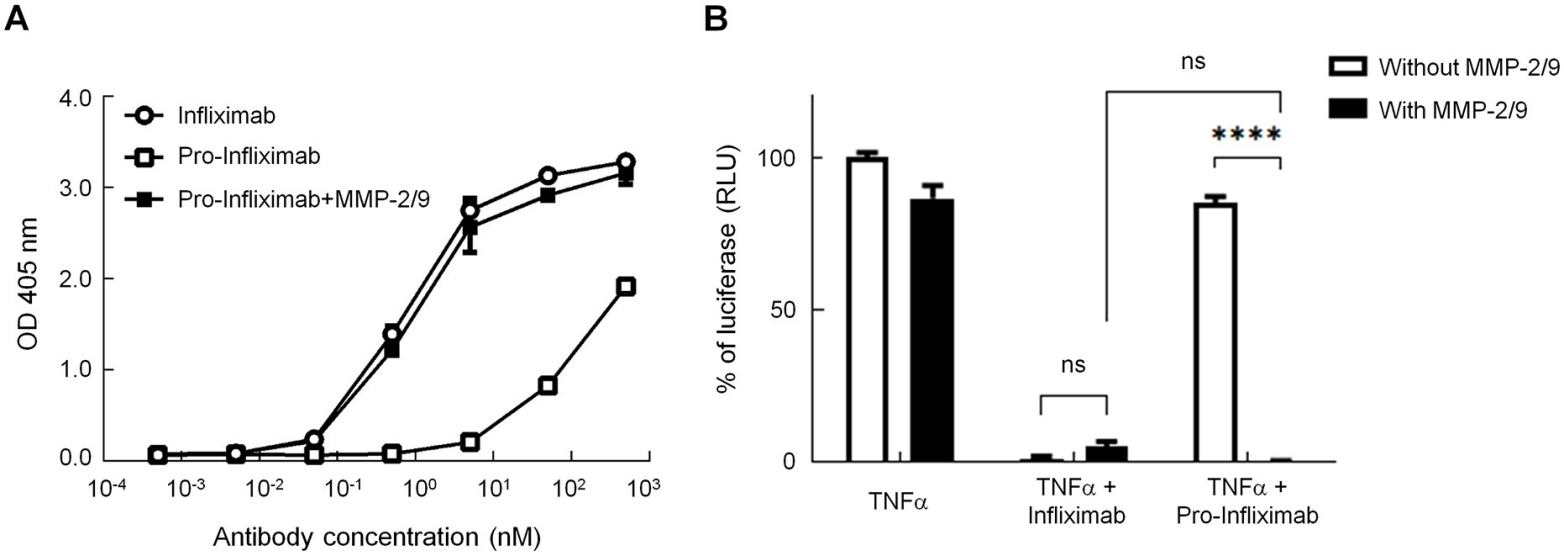
The Ab lock inhibits the binding ability and TNFα downstream signaling of Infliximab. (A) The binding ability of Infliximab (○), pro-Infliximab (□), and MMP-2/9–activated pro-Infliximab (■) was assessed by TNFα-based ELISA. (B) TNFα-neutralizing ability of pro-Infliximab and Infliximab with or without MMP-2/9 through luciferase NF-κB reporter assay. TNFα treatment was used as a control for reporter activity. The values are mean ± SEM, and the asterisks indicate a significant difference, ****P < 0.0001. Error bar: standard error of triplicate determinations. Underlying data can be found in S1 Data. Ab, antibody; MMP, matrix metalloproteinase; NF-κB, nuclear factor kappa B; NS, no significance; TNFα, tumor necrosis factor α.

We first evaluated the masking effect of the Ab lock by comparing the TNFα-binding ability of Infliximab and the pro-Infliximab by ELISA. The half-maximal effective concentration (EC_50_) of Infliximab and pro-Infliximab were 0.78 ± 0.05 nM and 308.60 ± 13.88 nM, respectively, showing that pro-Infliximab has an approximately 395-fold weaker antigen-binding ability than Infliximab (Fig 2A). Then, we investigated whether the Ab lock can be efficiently removed from pro-Infliximab and restore the TNFα-binding ability of pro-Infliximab after MMP-2/9 cleavage by western blot and ELISA. The results demonstrated that MMP-2/9 could completely remove the Ab lock from pro-Infliximab (S1 Fig), and the TNFα-binding ability of pro-Infliximab was gradually elevated in a time-dependent manner during MMP-2/9 treatment (S2 Fig). Further, after reacting with MMP-2/9 protease, the EC_50_ of pro-Infliximab was elevated from 308.60 ± 13.88 nM to 1.15 ± 0.18 nM and showed no significant difference (*P* = 0.89) as compared with control Infliximab. These results indicate that the antigen-binding ability of pro-Infliximab could be completely restored by MMP-2/9 cleavage. We next assessed the TNFα-neutralizing ability of MMP-2/9-activated pro-Infliximab by nuclear factor kappa B (NF-κB)-responsive reporter assay [18]. Fig 2B shows that the luciferase activity of pro-Infliximab–treated cells was similar to the TNFα treatment group. In contrast, MMP-2/9–activated pro-Infliximab significantly neutralized TNFα and reduced the luciferase activity (at least 283.3-fold as compared with inactive pro-Infliximab group) similar to that of the Infliximab-treated group (*P* > 0.05), suggesting that the MMP-2/9–activated pro-Infliximab could efficiently neutralize TNFα and block the downstream NF-κB signaling. Together, these results indicate that the Ab lock markedly masks the antigen-recognition ability of Infliximab, and inhibition of the biological function of pro-Infliximab could be completely restored after MMP-2/9 protease treatment.

### The Ab lock prevents the neutralizing effect of an anti-Id Ab on Infliximab

**Fig 3:**
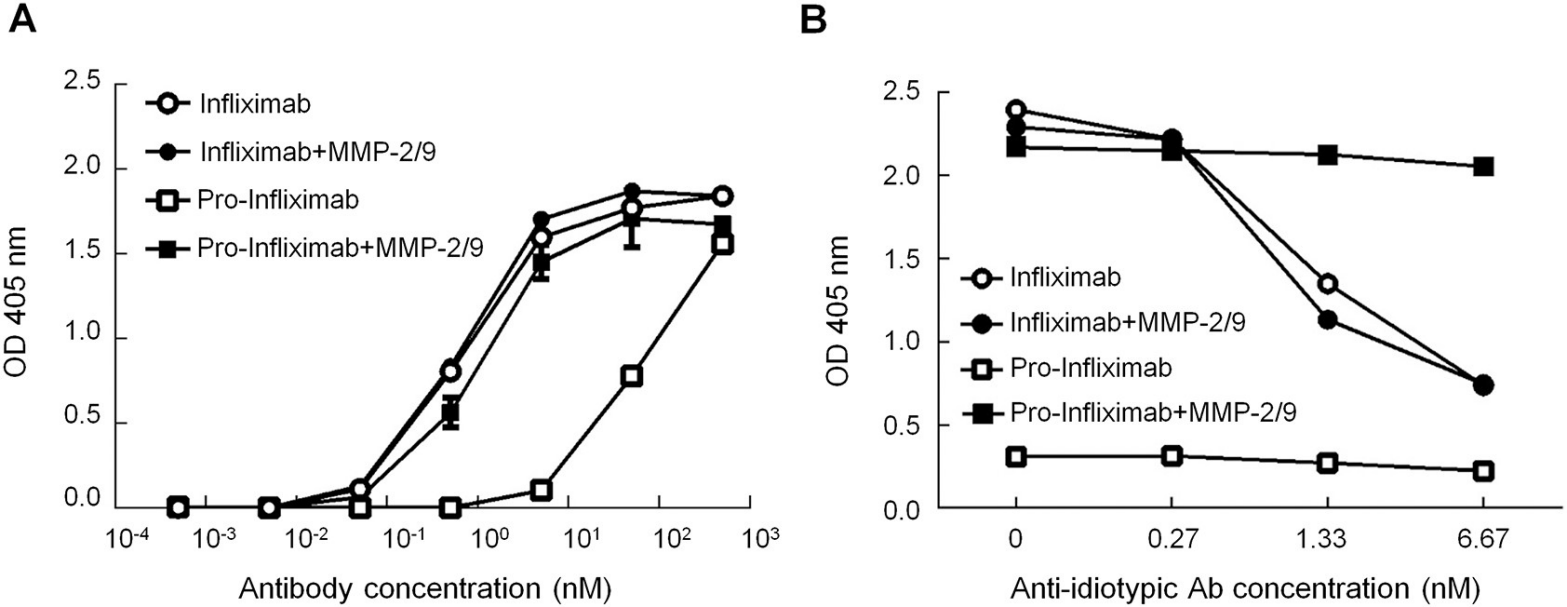
The Ab lock can prevent the response of the anti-Id Ab to Infliximab. (A) The anti-I-Id Ab–coated 96-well plate was incubated with different concentrations of Infliximab-biotin (○), pro-Infliximab-biotin (□), Infliximab-biotin preincubated with MMP-2/9 (●), or pro-Infliximab-biotin preincubated with MMP-2/9 (■), respectively, and then analyzed the neutralizing ability of anti-I-Id Ab by ELISA. (B) To further investigate the effect of anti-I-Id Abs on the restoration ability of TNFα binding of pro-Infliximab by protease. The Infliximab- or pro-Infliximab–coated plates were treated with different concentrations of anti-I-Id Abs. After removing unbound anti-I-Id Abs, we incubated the samples with or without MMP-2/9 and examined the TNFα-binding ability by TNFα-biotin and HRP-conjugated streptavidin. The values are mean ± SEM. Error bar: standard error of triplicate determinations. Underlying data can be found in S1 Data. Ab, antibody; anti-I-Id, anti-Infliximab idiotypic; HRP, horseradish peroxidase; MMP, matrix metalloproteinase; TNFα, tumor necrosis factor α.

Previous studies have reported that nearly half RA patients develop anti-Infliximab Abs, which are mostly known as anti-Id Abs, within the first year of Infliximab treatment [19, 20]. A high level of anti-Id Abs decreases the bioavailability and serum half-life of Infliximab, thereby impairing its therapeutic efficacy in RA patients [3, 21]. To evaluate whether the spatial-hindrance-based Ab lock could prevent pro-Infliximab bound by anti-Id Abs of Infliximab (anti-I-Id Abs), we immobilized the anti-I-Id Abs (Bio-Rad Laboratories, Redmond, WA, USA) on a plate and analyzed the binding ability of pro-Infliximab and Infliximab in the condition with or without MMP-2/9 by ELISA. Fig 3A indicates that the anti-I-Id Ab binding ability of pro-Infliximab (EC_50_ = 72.93 nM) was significantly lower than Infliximab (EC_50_ = 0.67 nM) and it can be completely restored after MMP-2/9 treatment, suggesting that pro-Infliximab possess approximately 108-fold weaker anti-I-Id Ab binding activity as compared to control Infliximab and the neutralizing effect of anti-I-Id Ab is reversible. To further investigate the effect of anti-I-Id Abs on the restoration ability of TNFα binding of pro-Infliximab by protease, we immobilized pro-Infliximab and Infliximab in plates and then treated them with different concentrations of anti-I-Id Abs. After removing unbound anti-I-Id Abs, we incubated the samples with or without MMP-2/9 and examined the TNFα binding ability by TNFα-biotin and horseradish peroxidase (HRP)-conjugated streptavidin. As shown in Fig 3B the TNFα binding ability of Infliximab and MMP-2/9-treated Infliximab were gradually decreased in a dose-dependent manner of anti-I-Id Abs. In contrast, the antigen binding ability of pro-Infliximab could be completely restored by treatment with MMP-2/9 and was comparable to control Infliximab without the presence of anti-I-Id Abs. These results suggest that the Ab lock was able to prevent the neutralizing effect of the anti-Id Abs of Infliximab whilst maintaining the restoration ability of pro-Infliximab by MMP-2/9.

### The Ab lock does not change the basic characteristics of Infliximab

**Fig 4:**
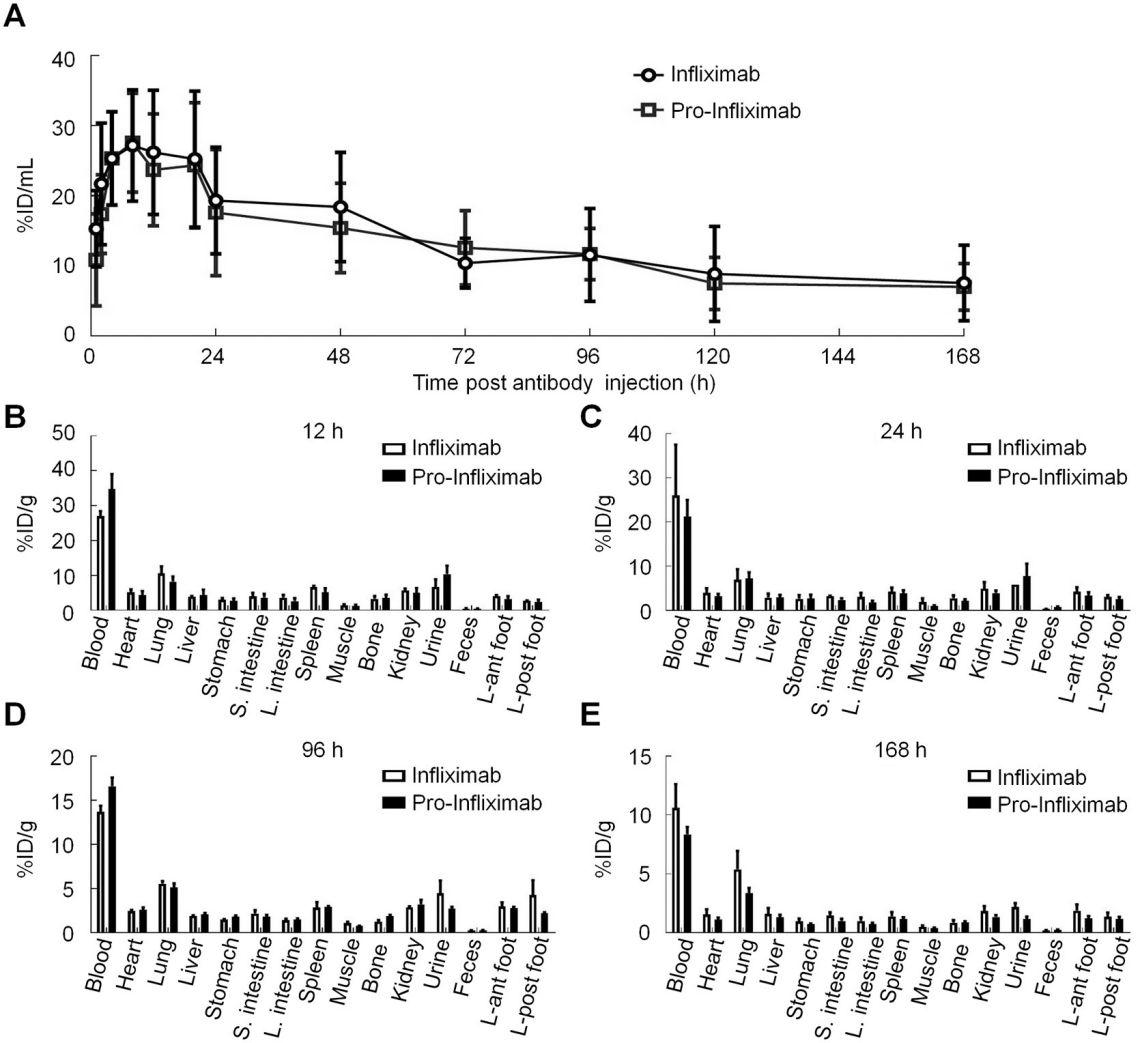
Evaluation of the PK properties and biodistribution of pro-Infliximab and Infliximab. The DBA/1J mice were intraperitoneally injected with ^131^I-labeled Infliximab (○) or ^131^I-labeled pro-Infliximab (□). The blood was collected at different time and the radioactivity was detected via the γ counter to measure the (A) PK properties of ^131^I-labeled Infliximab and ^131^I-labeled pro-Infliximab. The biodistribution of ^131^I-labeled Infliximab and ^131^I-labeled pro-Infliximab in several tissues and organs in DBA/1J mice at 12 h (B), 24 h (C), 96 h (D), and 168 h (E) after injection. PK properties and biodistribution are expressed as radioactivity %ID/g. Error bar: standard error of triplicate determinations. Underlying data can be found in S1 Data. DBA/1J, dilute brown non agouti; %ID/g, percent injected dose per gram; PK, pharmacokinetic.

To determine whether the Ab lock altered the basic properties of Infliximab, including the serum half-life and biodistribution, dilute brown non agouti (DBA/1J) mice were intraperitoneally injected with ^131^I-labeled pro-Infliximab or ^131^I-labeled Infliximab. The blood and organ specimens were collected, and the radioactivity was measured by a multichannel gamma counter at different time points after administration of radio-labeled Abs. As shown in Fig 4A, the average radioactivity concentrations of both pro-Infliximab and Infliximab in blood samples were gradually decreased and showed a similar curve profile post injection of Abs. The serum half-life of pro-Infliximab and Infliximab were 84.14 h and 83.98 h (Table 1). The pharmacokinetic (PK) analysis also demonstrated the exposure to pro-Infliximab as measured by the mean maximum serum concentration (C_max_), and the area under the plasma concentration (AUC) was similar to those of Infliximab. Additionally, both of the antibodies displayed lower clearance values (CI) and longer mean residence time (MRT) without significant differences. These results demonstrated that the Ab lock did not alter the PK characteristics of pro-Infliximab in circulation. Regarding biodistribution, we also observed that there was no significant difference (*P* > 0.05) in radioactivity (percent injected dose per gram [%ID/g]) between each corresponding organ derived from the pro-Infliximab–and Infliximab-injected mice at 12, 24, 96, and 168 h (Fig 4B–4E). These results suggest that the Ab lock did not affect the basic biological properties of pro-Infliximab and maintained PK and biodistribution similar to the control, Infliximab, in vivo.

**Table 1:** The Ab lock does not change the PK characteristics of Infliximab.

Test article	Cmax (%ID/mL)	AUC (h*%ID/mL)	CI (mL/h)	MRT (h)	Half-life (h)
Infliximab	27.2	2,280.1	0.03	128.9	84.0
Pro-Infliximab	27.6	2,147.5	0.03	128.3	84.1

**Abbreviations**: %ID/g, percent injected dose per gram; AUC, area under the plasma concentration; CI, clearance value; C_max_, mean maximum serum concentration; h, hour; MRT, mean residence time.

Additionally, plasma CI and MRT are shown.

### Site-specific activation of pro-Infliximab prevents RA progression

**Fig 5:**
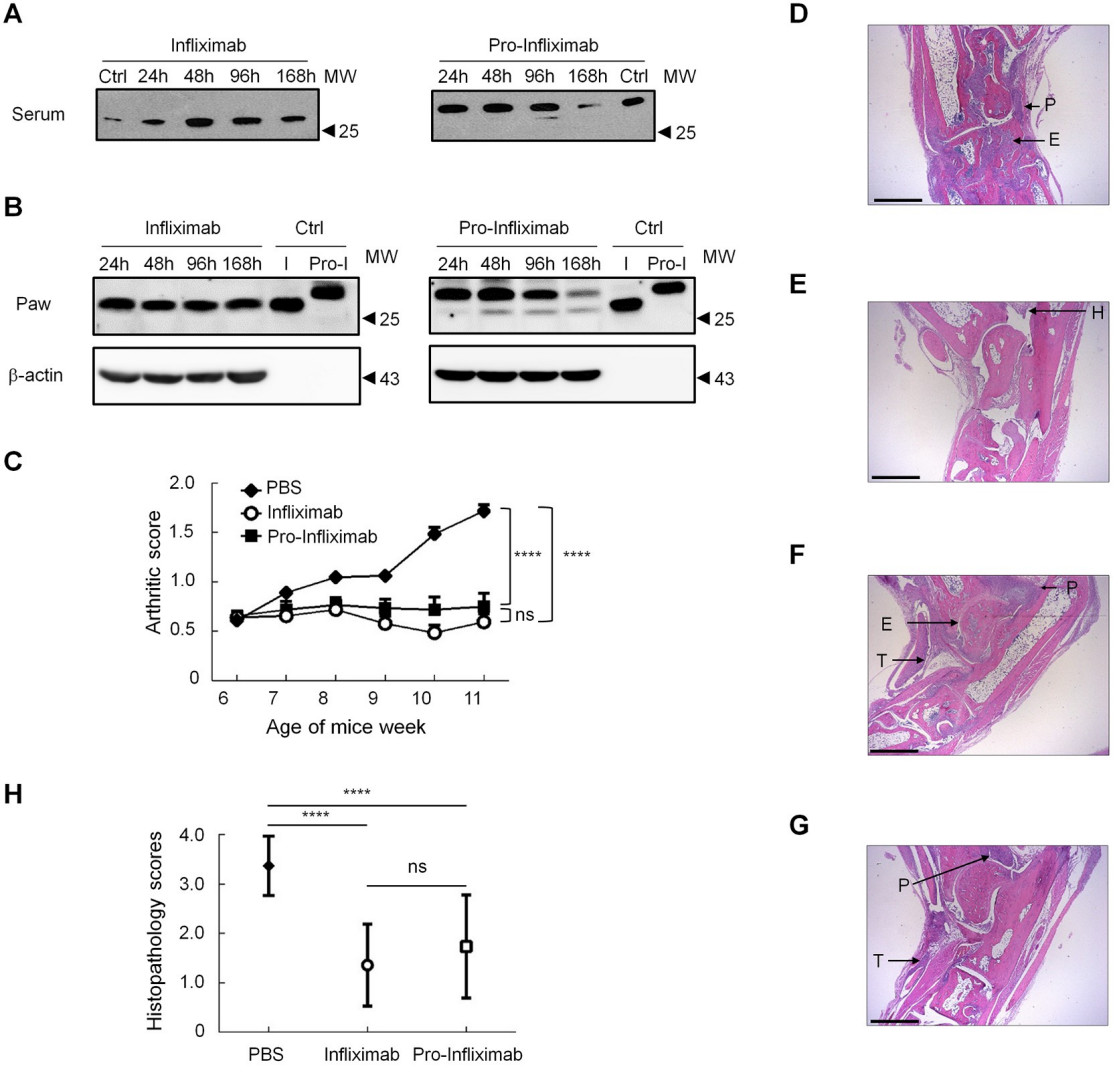
The pro-Infliximab is activated at the disease site and suppresses disease development. The hTNFα-transgenic 1006 mice were intraperitoneally injected with 50 μg Infliximab or pro-Infliximab. After 24, 48, 96, and 168 h, the blood and hind paw specimens were collected and using HRP-conjugated antihuman IgG Fab Ab for detecting the level of active and inactive pro-Infliximab in (A) serum and (B) joint tissue (paw) by western blot. The β-actin as internal control. I means Infliximab, pro-I means pro-Infliximab. The Tg197 mice (*n* = 8) were intraperitoneally injected with 10 mg/kg Infliximab (○), 10 mg/kg pro-Infliximab (■), or saline (◆). All mice were dosed twice weekly for 6 weeks. (C) Arthritic score of each group was monitored every week to measure the therapeutic efficacy. Representative HE stained sagittal tissue sections of the ankle joints in Tg197 mice after treatment with PBS (D), Infliximab (E, F), and pro-Infliximab (G) for 6 weeks. Representative histopathology images at 25× magnification. Bars = 1 mm. (H) The histopathology score of the paw in PBS, Infliximab, and pro-Infliximab–treated Tg197 mice. The values are mean ± SD, and the asterisks indicate a significant difference, *P < 0.01, **P < 0.001. Error bar: standard error of octuplicate determinations. Underlying data can be found in S1 Data. Ab, antibody; E, bone erosion; H, synovial hyperplasia; HE, hematoxylin–eosin; HRP, horseradish peroxidase; IgG; NS, no significance; P, pannus; T, tendonitis; Tg197 mice, hTNFα-transgenic mice; TNFα, tumor necrosis factor α.

To examine whether pro-Infliximab could be selectively activated in the RA region, hTNFα-transgenic 1006 mice, which can spontaneously develop moderate arthritis in the hind paws, were treated with pro-Infliximab or Infliximab. Blood and hind paw specimens were collected at different time points, and the molecular weight profile of pro-Infliximab and Infliximab were evaluated by western blot. Here, we used light chain molecular weight as an indicator of protease restoration. Fig 5A and 5B indicate that the light chain molecular weight (25.6 kDa) in the paw of Infliximab-treated mice was consistent with the molecular weight profile in the blood. By contrast, the light chain molecular weight (29.4 kDa) in the paws of pro-Infliximab–treated hTNFα-transgenic 1006 mice were only gradually converted into cleaved form (25.6 kDa) at 24, 48, 96 and 168 h, but there was only a slightly cleaved form observed in the blood at 168 h (Fig 5A and 5B). Further, pro-Infliximab was not activated by protease in lung tissue of pro-Infliximab–treated mice. And both Infliximab and pro-Infliximab were gradually degraded in a time-dependent manner after Ab treatment. However, there were no Infliximab or pro-Infliximab signals detected in colon and spleen tissue (S3 Fig). These results indicated that the Ab lock could be selectively released from pro-Infliximab in the RA region. To further investigate the preventive effect of pro-Infliximab and Infliximab on pathological progression of RA, hTNFα-transgenic mice (Tg197 mice), which can also spontaneously develop arthritis in the hind paws with deformed ankle joints at 4 weeks of age, were intraperitoneally injected with 10 mg/kg pro-Infliximab or Infliximab twice a week for 6 weeks by Biomedcode (Vari, Greece) animal facilities. The clinical score of pro-Infliximab–and Infliximab-treated Tg197 mice were significantly decreased as compared with control mice treated with normal saline (*P* < 0.0001, Fig 5C). Of note, there was no significant difference between the arthritis scores in either the pro-Infliximab–or Infliximab-treated groups (Fig 5C), suggesting that pro-Infliximab prevents RA in a similar manner to the control Infliximab. Both of these two Ab-treated mice gained more body weight than the normal saline-treated group (S4 Fig). In addition, the joint damage in hind paws was also evaluated by histopathologic analysis through hematoxylin–eosin (HE) staining. All joints were scored for synovitis, bone erosions, and cartilage degradation using a predefined scoring system [22]. Six weeks post-treatment, saline-treated Tg197 mice showed increased histopathologic scores for bone erosion, cartilage destruction, and pannus in hind paws (Fig 5D). In contrast, the synovial inflammation, bone erosions, and cartilage degradation were almost completely resolved in pro-Infliximab–and Infliximab-treated groups in comparison with the saline-treated group (*P* < 0.0001, Fig 5D–5G). Moreover, there was no significant difference in the histopathologic scores between the pro-Infliximab–and Infliximab-treated Tg197 mice (*P* = 0.29, Fig 5H). Collectively, these results indicate that pro-Infliximab could be specifically reactivated in the RA region and decreases RA progression with comparable efficacy to Infliximab.

### Selective restoration of pro-Infliximab reduces risk of opportunistic infection

**Fig 6:**
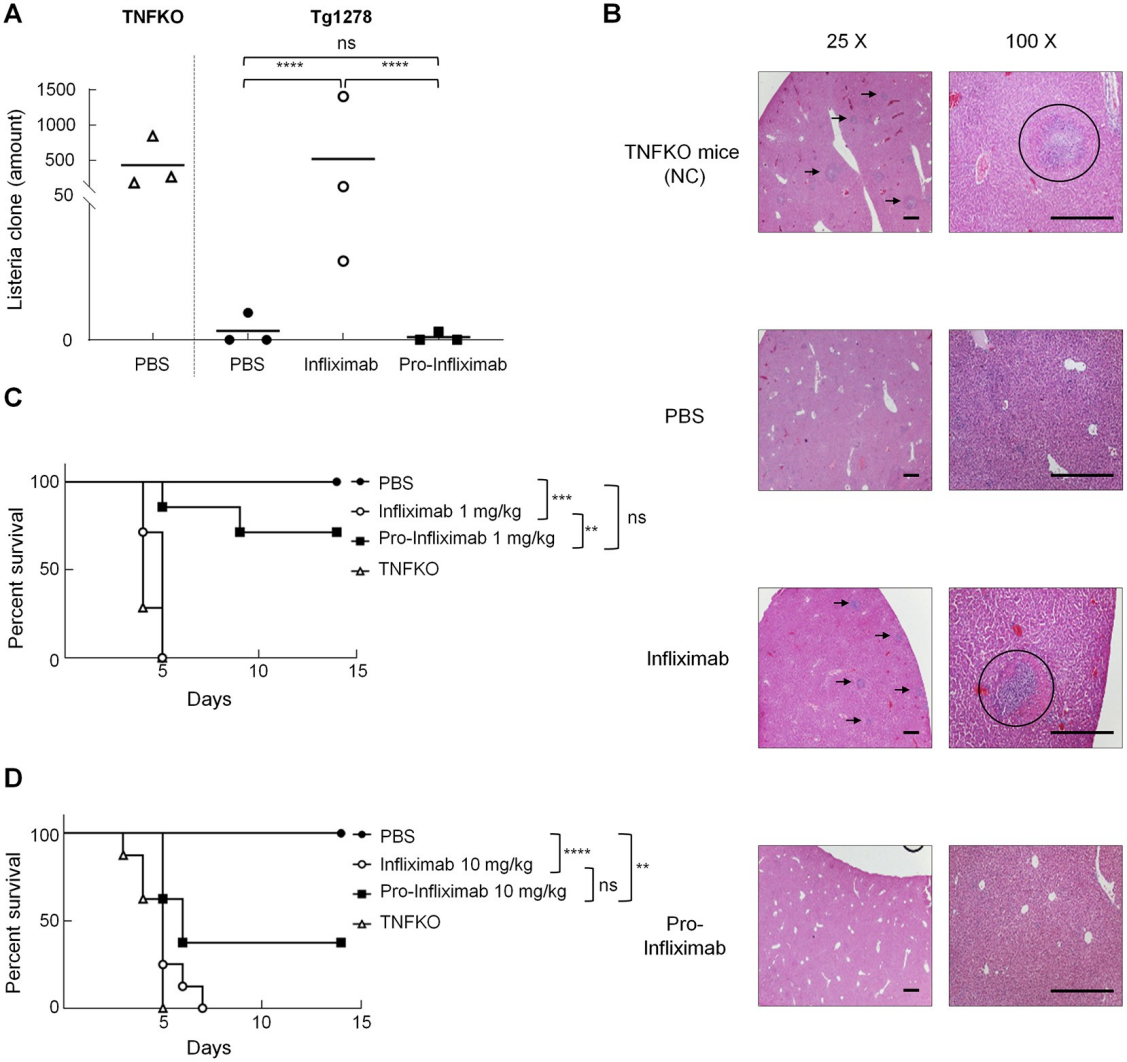
Comparison of the protective ability between pro-Infliximab and Infliximab on the host defense of TNFKO and Tg1278 TNFKO mice against *Listeria* infection. The Tg1278 mice (*n* = 3) were intraperitoneally injected with 1 and 10 mg/kg of pro-Infliximab, Infliximab, or control saline and challenged with lethal dosage of *L*. *monocytogenes* (×10^4^ CFU) 2 h later. The blood CFU counts (A) and representative HE staining (B) of liver tissues were tested to compare the host defense of *Listeria* infection. The arrows and circles represent the sites of infection identified as big well-shaped granuloma structures containing necrotic hepatocytes and inflammatory cells. Bars = 500 μm. (C and D) Survival rate after PBS, Infliximab. or pro-Infliximab treatment at low dose (1 mg/kg) (C) and high dose (10 mg/kg) (D) in *L*. *monocytogenes*-infected mice. The values are mean ± SEM and the asterisks indicate a significant difference, **P < 0.01, ***P < 0.001, ****P< 0.0001. Error bar: standard error of triplicate determinations. Underlying data can be found in S1 Data. CFU, colony-forming unit; HE, hematoxylin–eosin; NS, no significance; Tg1278 mice, hTNFα-transgenic mice; TNFα, tumor necrosis factor α; TNFKO, TNFα knockout.

Previous studies and an FDA report mentioned that long-term administration of Infliximab increases susceptibility to serious infections, even leading to hospitalization or death of RA patients. Listeriosis, caused by *L*. *monocytogenes*, is one of the most commonly reported opportunistic infections, which may cause sepsis and meningitis, in Infliximab-treated RA patients [23, 24]. To investigate whether the regionally restored pro-Infliximab reduces the risk of opportunistic infection during Ab treatment, hTNFα-transgenic mice (Tg1278 mice), which only express normally regulated hTNFα but lack mTNFα [25], were intraperitoneally injected with 1 and 10 mg/kg of pro-Infliximab, Infliximab, or control saline before being challenged with a lethal dose of *L*. *monocytogenes* (×10^4^ colony-forming unit [CFU]). Blood and liver tissue specimens were collected to analyze the CFUs of *L*. *monocytogenes* and the histopathologic phenotype, respectively. The number of *Listeria* CFUs in the blood from pro- Infliximab–or saline-treated mice was significantly lower (****P < 0.0001) than those from blood samples from Infliximab-treated mice and TNFα knockout (TNFKO) mice (Fig 6A), indicating that systemic TNFα is not recognized by pro-Infliximab and protects the host from *L*. *monocytogenes* infection. On the other hand, the histopathologic data of liver tissue from pro-Infliximab–treated mice exhibited fewer infectious foci (1–2 per microscope field) and infiltrated immune cells in comparison with the Infliximab-treated groups (4–5 per microscope field, Fig 6B). The overall survival rate of pro-Infliximab–treated mice was also significantly longer than Infliximab-treated Tg1278 mice and untreated TNFKO mice (mTNFα: −/−, hTNFα: −/−) in 1 and 10 mg/kg dosage administration (Fig 6C and 6D) and showed 71.4% and 37.5% survival until the end of the study, respectively. In contrast, mice treated with a low dose (1 mg/kg) or high dose (10 mg/kg) of Infliximab showed 100% lethality within 7 days similar to *Listeria*-challenged TNFKO mice, indicating that the systemic blockage of TNFα by Infliximab destroys the TNF-dependent host defense mechanism and aggravates infection of *Listeria* in the host. Together, these results suggest that pro-Infliximab may not affect systemic TNFα during circulation thoroughly, thus keeping its biological functions and preventing the undesirable blocking effect of TNF-dependent host defense mechanism, thereby maintaining part of host immunity during RA treatment.

## Discussion

We have successfully developed an RA region, highly selective pro-Infliximab using an autologous hinge domain, which can significantly mask the TNFα-binding ability of Infliximab. This pro-Infliximab is selectively activated by disease-associated MMP-2/9 in the RA region and shows similar PKs and therapeutic efficacy as Infliximab without dysregulating systemic immunity against *Listeria* infection in vivo. Additionally, the spatial hindrance–based Ab lock (Hinge) can prevent the neutralizing effect on antigen-binding ability of Infliximab from anti-Id Ab, which is a major problem in Ab-based therapy after repeating administration of Ab drugs. We believe that the MMP-cleavable and efficient Ab lock can significantly increase the selective reaction of Infliximab at the disease site and reduce the on-target toxicities of Infliximab during systemic circulation, thereby showing potential to improve the quality of life of RA patients.

Development of an Ab lock with high masking efficiency is important to enhance the selectivity of Ab drugs at the disease region long term. Unfortunately, no efficient strategy has hitherto been developed for improving selective activation of Infliximab at the RA region. Recently, Chen and colleagues attempted to solve this problem by creating a protease-activation pro-Infliximab with an inhibitory domain (latency-associated peptide [LAP]) that can be removed by proteases. [26] However, the LAP domain could only mask 1.86-fold of the TNFα-binding ability of the original Infliximab and significantly increased the molecular weight (MW) of pro-Infliximab (approximately 240 kDa) [26]. The poor masking efficiency and high MW of this pro-Infliximab may be insufficient to prevent the on-target toxicities and affect its PKs, respectively, during systemic administration of pro-Infliximab in RA patients. Additionally, the productive efficiency of this pro-Infliximab was significantly affected by the LAP domain and retained only 33% productive yield as compared with original Ab, [26] which led to difficulty in obtaining enough pro-Infliximab to investigate the PK, biodistribution, therapeutic efficacy, and on-target toxicities in an RA mouse model. In our study, we demonstrated that the Ab lock exhibits a 369-fold masking effect and only slightly increases the MW of Infliximab (approximately 160 kDa) (Fig 1A). The high masking efficiency and low MW means the Ab lock did not affect the productive yield and basic biological properties of pro-Infliximab and maintained the PK and biodistribution as control Infliximab in vivo (Fig 4). Our results also suggest that the pro-Infliximab has similar therapeutic efficacy and significantly decreases the risk of opportunistic infection in the RA mouse model as compared with Infliximab (Figs 5 and 6). Collectively, this is the first study to prove the concept that increasing the selectivity of the Ab drug at the disease site can reduce the risk of adverse effects whilst maintaining its therapeutic efficacy. We believe that the Ab lock is a novel and more suitable masking strategy for improving the selective activation of Infliximab at the RA region without altering its basic characteristics.

Blocking the binding of anti-Id Abs that influence the effectiveness of Ab drugs in RA treatment is very important because antidrug antibodies (ADAs, and mostly anti-Id Abs) have been reported to play a major role in causing a secondary response failure, in which the effectiveness of Ab drugs for RA therapy is lost over time despite a good initial response [27]. For example, 67% of patients with RA are treated with Adalimumab (fully human anti-TNFα Ab; Humira) developed ADAs within the first 28 weeks of treatment [28]. The antibodies against Infliximab can be detected in sera from 7% to 53% of Infliximab-treated RA patients [27]. The appearance of ADAs was associated with reduced serum concentrations of Infliximab and Adalimumab, decreased clinical response, and increased the risk of adverse events [19, 21]. Studies have also reported that 63% of patients with RA developed the anti-Campath (anti-CD52) Ab, with reduced therapeutic efficacy. [29] Here, we demonstrated that the spatial hindrance–based Ab lock can protect the complementarity-determining region (CDR) loop to prevent the response of the applied anti-Id Ab to Infliximab (anti-I-Id Ab); the binding of the anti-I-Id Ab to pro-Infliximab was 108-fold weaker than that to Infliximab (Fig 2). We also found that the binding ability of activated pro-Infliximab was not influenced by the application of the anti-I-Id Ab. These results suggest that with the Ab lock the pro-Infliximab has the potential to replace the Infliximab or serve as a second-line drug for RA patients with anti-I-Id Ab. Concerns may be raised that the administration of pro-Ab could induce the production of anti-hinge Ab. We also analyzed the immunogenicity of pro-Infliximab by treating synthetic MMP-2/9 substrate sequence, Infliximab, or pro-Infliximab, respectively, to differentiated primary dendritic cells that coculture with CD4^+^ T cells (S5 Fig, the detailed experimental procedure can be found in S1 Text). The result indicated that there was no significant difference of CD4^+^ T cell proliferation between MMP-2/9 substrate sequence–treated, Infliximab-treated, pro-Infliximab–treated group, and control group. Moreover, IgG is the most abundant Ab in normal human serum, for about 10%–20% of plasma protein [30]. Furthermore, the IgG1 hinge domain is plentiful and derived from the Abs themselves. The autologous hinge should be the most appropriate Ab lock to form low immunogenicity pro-Ab drug.

TNFα-neutralizing Abs have been widely used to treat several autoimmune and immune-mediated disorders, such as RA [31], psoriasis [32, 33], and Crohn disease [34]. Among these diseases, proteases are attractive disease markers [35–37] and play important roles in various disease progression due to their high specificity and local activity [38]. The changeable design of protease substrates in the pro-Ab allows an Ab lock that can be widely applied to other diseases smoothly. For example, Infliximab is also used in the treatment of plaque psoriasis; we can replace the MMP-2/9 substrate linker with a marapsin substrate linker based on marapsin, which is a strongly up-regulated protease in psoriatic lesions [39]. In addition, there are several alternative Ab drugs for slowing the progression of RA by interrupting the immune process that promotes inflammation and joint damage. For instance, Tocilizumab (TCZ) is a humanized monoclonal Ab that acts against interleukin-6 receptor (IL-6R) for preventing the binding of proinflammatory cytokines (i.e., IL-6) to immune cells and is used for the treatment of moderate to severe RA disease [40]. Secukinumab (AIN457) was also developed for neutralizing IL-17A, which is a proinflammatory cytokine mainly secreted from T helper cells (Th-17) and currently in Phase III clinical trials for evaluation of its therapeutic efficacy and safety in RA treatment [41]. However, long-term inhibition of IL-6– or IL-17A–induced proinflammatory effects by Ab drugs may hamper the immunity of RA patients and increase the risk of adverse events such as serious tuberculosis infection [42] or fungal infection [43]. The spatial hindrance–based Ab lock is expected to be able to mask the antigen-binding site of a broad range of Ab drugs due to its highly conserved structure. Actually, we have also proved that the Ab lock could widely and significantly inhibit the antigen-binding ability of Ab drugs with different antigen specificities. For example, pro-anti-IL6 receptor Ab has an approximately 50.3-fold weaker antigen-binding ability than pro-anti-IL6 receptor Ab alone. (S6 Fig, the detailed experimental procedure can be found in S1 Text.) We believe that the universal Ab lock with changeable design of protease substrate can be applied to any alternative Ab drugs that are used for RA or even other disease treatment to improve their selective reactivity and clinical benefit.

In conclusion, we found that an autografted hinge can convert Infliximab into prodrug forms (pro-Infliximab) to improve the selectivity and reduce on-target toxicities of Infliximab. The pro-Infliximab has the following advantages: (1) it can be selectively activated in the RA region after cleaving by overexpressed protease; (2) it does not dysregulate the systemic immunity of TNFα; (3) it does not change the productive yield, biodistribution, and PKs of the original Ab; (4) it may have the lowest immunogenicity because the hinge normally exists in human serum; and (5) it can prevent the neutralizing effects of anti-Infliximab idiotypic Ab and aid the realization of the full potential of Infliximab. We expect that this novel strategy for improving selective activation of Infliximab can significantly improve the quality of life in RA patients and cause revolutionary effect on the development industry of Ab drugs in the future.

## Materials and methods

### Ethics statement

DBA/1J, 1006 mice (mTNFα: +/+, hTNFα: +/−) were purchased from the Jackson Laboratory (Bar Harbor, ME, USA) and Taconic (Germantown, NY, USA), respectively.

Animal experiments were carried out in accordance with institutional guidelines and approved by the Animal Care and Use Committee of Kaohsiung Medical University, Kaohsiung, Taiwan (IACUC:104045). Tg197 mice (mTNFα: +/+, hTNFα: +/−), Tg1278 TNFKO (mTNFα: −/−, hTNFα: +/−), and TNFKO (mTNFα: −/−, hTNFα: −/−) mice were housed, maintained, and operated at Biomedcode (Vari, Greece) animal facilities, according to standard operating procedures. The Test Facility is accredited by the Hellenic Republic Prefecture of East Attica, Veterinary Service and Fishery Department and registered with the Ministry of Agriculture to conduct research in laboratory animals. All of the conditions of testing conformed to the Presidential Decree No. 160/1991 Governmental Gazette No. A’ 64 applicable in Greece, which is the implementation of the EEC Directive 86/609/EEC, and were approved by the Veterinary Service Management of the Hellenic Republic Prefecture of East Attica (Approval license protocol No. 3720, 03/07/2013). The protocol was reviewed and approved by the Institutional Animal Ethical Committee at the Biomedical Sciences Research Centre “Al. Fleming” for compliance with regulations (Approval license No. 1125 on 23 February 2016). Due to the use of infectious material (*L*. *monocytogenes*), the study was performed in the Biosafety Level 2 facility of BSRC Al. Fleming, according to the relevant safety rules and regulations.

### Pro-Ab construction, expression, and purification

The complementary DNA coding for the heavy and light chains of Infliximab were cloned through assembly PCR. Human IgG1 hinge sequences were obtained from the National Center for Biotechnology Information. The hinge-encoding sequences, GGGGS linker, and MMP-2/9 substrate–encoding sequences (GPLGVR) [44] were introduced upstream of the light chain and heavy chain of Infliximab to generate pro-Infliximab. Infliximab or pro-Infliximab production were through the Expi293 Expression System (Thermo Fisher Scientific, Waltham, MA, USA) and purified by Protein A-Sepharose (GE Healthcare, Milwaukee, WI, USA).

### Comparison of the binding ability of pro-Infliximab with or without MMP-2/9 treatment

To determine the binding kinetics (EC_50_) of pro-Infliximab and Infliximab, TNFα was coated onto 96-well plates and blocked with 5% skim milk. Infliximab or pro-Infliximab was incubated with or without 20 μg/mL of MMP-2/9 (type IV collagenase, Sigma-Aldrich, St. Louis, MO, USA) in DMEM/0.05% BSA (pH 7.4) for 1 h at 37 °C before the reaction was terminated by BCS. All of the samples were added onto the plates at the given concentrations (500–0.001 nM) for 1 h at RT. After washing, the wells were incubated with HRP-goat antihuman IgG Fcγ Ab for 1 h at RT, and detection was performed by the addition of ABTS containing 30% H_2_O_2_ (Sigma-Aldrich). The binding ability was quantified through absorbance detection at 405 nm.

### Neutralization of TNFα signal by pro-Infliximab

Human embryonic kidney cell line (HEK293) cells were transiently transfected with the luciferase reporter plasmid pNF-κB-Luc [45] (BD Biosciences Clontech, Palo Alto, CA, USA), internal control Renilla-Luc reporter plasmid, or positive control pFC-MEKK plasmid with TransIT-LT1 reagent (Mirus Bio, Madison, WI) for 6 h. After the transfection procedure, the medium was replaced with TNFα (20 ng/mL, Sigma-Aldrich) and incubated with Infliximab, pro-Infliximab, MMP-2/9 preincubated Infliximab, or MMP-2/9 preincubated pro-Infliximab for 1 h at 37 °C. After removing from the medium, the cells were cultured for 16 h at 37 °C, and cell extracts from each sample were measured using the dual-luciferase reporter assay system (Promega, Madison, WI, USA) according to the manufacturer’s protocol.

### Evaluation of the binding ability of anti-Id Ab to pro-Infliximab

Anti-Id Ab (0.3 μg/mL, Bio-Rad Laboratories, Redmond, WA, USA) was coated onto 96-well plates and blocked with 5% skim milk. Infliximab-biotin or pro-Infliximab–biotin was incubated with or without 25 mg of MMP-2/9 in DMEM/0.05% BSA (pH 7.4) for 1 h at 37 °C before the reaction was terminated by BCS. All the samples were added onto the plates at the given concentrations for 1 h at RT. After washing, the plate was sequentially incubated with HRP-conjugated streptavidin (Jackson ImmunoResearch Laboratories). The detection was performed by the addition of ABTS containing 30% H_2_O_2_. The binding ability was quantified through absorbance detection at 405 nm.

To investigate the effect of anti-Id Ab on the restoration ability of TNFα binding of pro-Infliximab by protease, 96-well plates were coated with Infliximab and pro-Infliximab for 2 h at 37 °C and blocked with 5% skim milk. The plates were incubated with different concentrations (8–1,000 ng/mL) of anti-Id Ab for 1 h, then unbound anti-Id Ab was removed with PBST. Each sample was incubated with or without 20 μg/mL of MMP-2/9 for 1 h. After washing, each sample was incubated with TNFα-biotin for 1 h. The plates were washed with PBS and sequentially incubated with HRP-conjugated streptavidin. The procedures were performed as described previously.

### Measurement of the serum half-life and biodistribution in vivo

DBA/1J mice (*n* = 5) were injected intraperitoneally with 50 μCi ^131^I-labeled pro-Infliximab or 50 μCi ^131^I-labeled Infliximab. Whole blood was collected via the tail vein at different time points. The organ specimens were harvested at 12, 24, 96, and 168 h points postadministration of radio-labeled antibodies. The blood and tissue were weighed on an analytical balance and assayed for radioactivity in a multichannel gamma counter. The initial and terminal half-life of the probes were estimated by fitting the data to a two-phase exponential decay model with Prism software (Graphpad Software, San Diego, CA).

### Removal of the inhibitory domain from pro-Infliximab through MMP-2/9 treatment or MMP-2/9 in the disease region

Human TNFα-transgenic 1006 mice [46] (mTNFα: +/+, hTNFα: +/−) can develop spontaneous arthritis at about 7 or 8 weeks of age. Fourteen-week-old 1006 mice with moderate disease were intraperitoneally injected with 50 μg/mouse Infliximab or pro-Infliximab. After 24, 48, 96, and 168 h, the mice were sacrificed and blood, colon, lung, spleen, and hind paw specimens were collected. All tissues were homogenized and centrifuged to collect the supernatant and the light-chain MW profile of pro-Infliximab and Infliximab in the hind paw, and serum samples were than evaluated by western blot with HRP-αhIgG Fab Ab (Jackson ImmunoResearch Laboratories, West Grove, PA, USA). β-actin was detected by rabbit anti β-actin Ab (Cell Signaling Technology; Boston, Massachusetts, USA) and HRP-conjugated anti-rabbit IgG (H+L) Ab (Invitrogen, Carlsbad, CA, USA).

### Therapeutic efficacy of pro-Infliximab for rheumatoid arthritis in vivo

The 6-week-old Tg197 mice [47] (*n* = 8) were intraperitoneally injected with 10 mg/kg Infliximab, 10 mg/kg pro-Infliximab, or saline. All mice were dosed twice weekly for 6 weeks. The therapeutic efficacy of each group was monitored every week and evaluated using weight measurements and clinical scores. Clinical scores were based on a previously described scoring system [48]. After treatment for 6 weeks, all mice were sacrificed, and the hind ankle joints were removed for histology. The mouse paws were removed and fixed with formalin buffer for HE staining, and the histopathologic score was evaluated microscopically, as described previously [22], using a modified scoring system.

### Comparison of on-target toxicities of pro-Infliximab and Infliximab in vivo

The Tg1278 mice, which can only express normally regulated hTNFα but lack mTNFα [25], were intraperitoneally injected with 1 and 10 mg/kg of pro-Infliximab, Infliximab, or control saline and challenged with lethal dosage of *L*. *monocytogenes* (×10^4^ CFU) 2 h later. After 48 h, the three mice per group were sacrificed, and blood samples were collected for *Listeria* CFU determination while liver tissues were harvested for histopathological assessment; the other mice (*n* = 7) were monitored daily, and their survival was recorded for a total period of 2 weeks.

### Statistical analysis

Data are presented as mean ± SEM or SD. The protective effect of pro-Infliximab and Infliximab on pathological progression of RA and the mean body weight of Tg197 mice were analyzed with two-tailed Mann–Whitney test or unpaired *t* test, respectively, and the number of *Listeria* CFUs in the blood specimens was analyzed with F-test to compare the statistical significance of the differences between the controls and samples. Statistical analysis was performed using the GraphPad Prism v.6, and data were considered significant at a *P* value of less than 0.05.

## Supporting information

S1 Fig MW of pro-Infliximab and Infliximab with MMP-2/9 for different lengths of time. To investigate whether the Ab lock can be efficiently removed from pro-Infliximab and restore the antigen-binding ability of pro-Infliximab after MMP-2/9 cleavage, we incubated pro-Infliximab and Infliximab with MMP-2/9 for different time periods and evaluated the MW profile of Ab fragments by western blot. The results showed that the heavy-chain (58 kDa) and light-chain (29.4 kDa) molecular weight of pro-Infliximab was converted into a profile similar to the control Infliximab (55 kDa for heavy chain and 25.6 kDa for light chain) after treatment with MMP-2/9 within 60 min, demonstrating that MMP-2/9 could completely remove the Ab lock from pro-Infliximab. Ab, antibody; MMP, matrix metalloproteinase; MW, molecular weight.
(TIF)

S2 Fig TNFα binding of pro-Infliximab and Infliximab with MMP-2/9 for different lengths of time. The TNFα-binding ability of pro-Infliximab was gradually elevated in a time-dependent manner during MMP-2/9 treatment and finally restored to a level similar to the control Infliximab. MMP, matrix metalloproteinase; TNFα, tumor necrosis factor α.
(TIF)

S3 Fig The activation of pro-Infliximab in peripheral organ. hTNFα-transgenic 1006 mice were intraperitoneally injected with 50 μg Infliximab or pro-Infliximab. After 24, 48, 96, and 168 h, the (A) lung, (B) colon, and (C) spleen tissue were collected using HRP-conjugated anti-human IgG Fc Ab for detecting the level of active and inactive pro-Infliximab by western blot. The β-actin as internal control. Ab, antibody; Fc, fragment crystallizable; HRP, horseradish peroxidase; IgG, immunoglobulin; TNFα, tumor necrosis factor α.
(TIF)

S4 Fig Effect of pro-Infliximab and Infliximab on the mean body weight of Tg197 mice. By the end of the study (11 weeks of age), the mean body weights of all groups treated twice weekly from week 6 were as follows: PBS = 18.10 ± 1.54 g, Infliximab 10 mg/kg = 24.41 ± 1.37 g, and pro-Infliximab 10 mg/kg = 22.57 ± 1.64 g. Error bars indicate standard error of the mean. Tg197 mice, hTNFα-transgenic mice; TNFα, tumor necrosis factor α.
(TIF)

S5 Fig Immunogenicity of human immune cells to Infliximab, pro-Infliximab, and MMP-2/9 SL. We cocultured dendritic cells differentiated from human PBMCs with autologous CD4^+^ T cells and stimulated with control medium (represented as DC+T), PHA (as positive control), Infliximab, pro-Infliximab, or MMP-2/9 SL, respectively, for 5 days. Then, we detected the proliferation of CD4^+^ T cells by ATPlite Luminescence Assay kit (Perkin Elmer). Bars, SD. CPM, counts per minute; MMP, matrix metalloproteinase; PBMC, peripheral blood mononuclear cell; PHA, phytohemagglutinin; SL, substrate linker.
(TIF)

S6 Fig The Ab lock inhibits the IL-6R–binding ability of pro-anti-IL6 receptor Ab. The IL-6R–binding ability were analyzed by antigen based ELISA. The EC_50_ of anti-IL6 receptor Ab, pro-anti-IL6 receptor Ab, and MMP-2/9–activated pro-anti-IL6 receptor Ab were 1.77 nM, 88.97 nM, and 2.827 nM. Ab, antibody; EC_50_, half-maximal effective concentration; IL-6R, interleukin-6 receptor; MMP, matrix metalloproteinase.
(TIF)

S1 Text This file contains supplemental methods and references. (DOCX)

S1 Data This file contains the raw data presented in figures in the main manuscript (Figs 2–6) and supplemental figures (S2, S4, S5 and S6 Figs). (XLSX)

## Abbreviations

%ID/g: percent injected dose per gram
Ab: antibody
ADA: antidrug antibody
anti-Id: anti-idiotypic
AUC: area under the plasma concentration
CDR: complementarity-determining region
CFU: colony-forming unit
CI: clearance value
C_max_: mean maximum serum concentration
DBA/1J: dilute brown non agouti
EC_50_: half-maximal effective concentration
FDA: Food and Drug Administration
HBsAg: HBV surface antigen
HBV: hepatitis B
HE: hematoxylin–eosin
IgG1: immunoglobulin G1
IL-6R: interleukin-6 receptor
LAP: latency-associated peptide
MMP: matrix metalloproteinase
MRT: mean residence time
MW: molecular weight
NF-κB: nuclear factor kappa B
PK: pharmacokinetic
RA: rheumatoid arthritis
TB: tuberculosis
TCZ: Tocilizumab
Tg197 mice: hTNFα-transgenic mice
TNFα: tumor necrosis factor α
TNFKO: TNFα knockout

## References

[1] Segal B, Rhodus NL, Patel K. Tumor necrosis factor (TNF) inhibitor therapy for rheumatoid arthritis. Oral surgery, oral medicine, oral pathology, oral radiology, and endodontics. 2008;106(6):778–87. 10.1016/j.tripleo.2008.07.025 . 18930662

[2] Monaco C, Nanchahal J, Taylor P, Feldmann M. Anti-TNF therapy: past, present and future. International immunology. 2015;27(1):55–62. 10.1093/intimm/dxu102 . 25411043 PMC4279876

[3] Perdriger A. Infliximab in the treatment of rheumatoid arthritis. Biologics: targets & therapy. 2009;3:183–91. . 19707407 10.2147/btt.2009.3099 PMC2726073

[4] Administration. FaD. Remicade (Infliximab): Highlights of prescribing information.: Food and Drug Administration.; 2011 https://www.accessdata.fda.gov/drugsatfda_docs/label/2011/103772s5281lbl.pdf.

[5] Administration FaD. Humira (Adalimumab): Highlights of prescribing information. 2008 https://www.accessdata.fda.gov/drugsatfda_docs/label/2008/125057s114lbl.pdf.

[6] Ferreira I, Isenberg D. Vaccines and biologics. Annals of the rheumatic diseases. 2014;73(8):1446–54. 10.1136/annrheumdis-2014-205246 . 24845388

[7] Botha-Scheepers SA, Sarembock B. Infections in the management of rheumatic diseases: An update. South African medical journal = Suid-Afrikaanse tydskrif vir geneeskunde. 2015;105(12):1076 . 26933722 10.7196/samj.2015.v105i12.10220

[8] Mori S, Fujiyama S. Hepatitis B virus reactivation associated with antirheumatic therapy: Risk and prophylaxis recommendations. World journal of gastroenterology. 2015;21(36):10274–89. 10.3748/wjg.v21.i36.10274 . 26420955 PMC4579875

[9] Vigano M, Degasperi E, Aghemo A, Lampertico P, Colombo M. Anti-TNF drugs in patients with hepatitis B or C virus infection: safety and clinical management. Expert opinion on biological therapy. 2012;12(2):193–207. 10.1517/14712598.2012.646986 . 22188392

[10] Mercer LK, Askling J, Raaschou P, Dixon WG, Dreyer L, Hetland ML, et al Risk of invasive melanoma in patients with rheumatoid arthritis treated with biologics: results from a collaborative project of 11 European biologic registers. Annals of the rheumatic diseases. 2017;76(2):386–91. 10.1136/annrheumdis-2016-209285 . 27307502 PMC5284347

[11] Singh JA, Cameron C, Noorbaloochi S, Cullis T, Tucker M, Christensen R, et al Risk of serious infection in biological treatment of patients with rheumatoid arthritis: a systematic review and meta-analysis. Lancet. 2015;386(9990):258–65. 10.1016/S0140-6736(14)61704-9 . 25975452 PMC4580232

[12] Mariette X, Tubach F, Bagheri H, Bardet M, Berthelot JM, Gaudin P, et al Lymphoma in patients treated with anti-TNF: results of the 3-year prospective French RATIO registry. Annals of the rheumatic diseases. 2010;69(2):400–8. 10.1136/ard.2009.117762 . 19828563 PMC2925048

[13] Calderon-Gonzalez R, Frande-Cabanes E, Bronchalo-Vicente L, Lecea-Cuello MJ, Pareja E, Bosch-Martinez A, et al Cellular vaccines in listeriosis: role of the Listeria antigen GAPDH. Frontiers in cellular and infection microbiology. 2014;4:22 10.3389/fcimb.2014.00022 . 24600592 PMC3930854

[14] Hatzara C, Hadziyannis E, Kandili A, Koutsianas C, Makris A, Georgiopoulos G, et al Frequent conversion of tuberculosis screening tests during anti-tumour necrosis factor therapy in patients with rheumatic diseases. Annals of the rheumatic diseases. 2015;74(10):1848–53. 10.1136/annrheumdis-2014-205376 . 24854354

[15] Chung SJ, Kim JK, Park MC, Park YB, Lee SK. Reactivation of hepatitis B viral infection in inactive HBsAg carriers following anti-tumor necrosis factor-alpha therapy. The Journal of rheumatology. 2009;36(11):2416–20. 10.3899/jrheum.081324 . 19797507

[16] Schioppo T, Ingegnoli F. Current perspective on rituximab in rheumatic diseases. Drug design, development and therapy. 2017;11:2891–904. 10.2147/DDDT.S139248 . 29042750 PMC5633295

[17] Administration. FaD. Rituxan (Rituximab): Highlights of prescribing information.: Food and Drug Administration; 2018. https://www.accessdata.fda.gov/drugsatfda_docs/label/2012/103705s5367s5388lbl.pdf.

[18] Kothary N, Diak IL, Brinker A, Bezabeh S, Avigan M, Dal Pan G. Progressive multifocal leukoencephalopathy associated with efalizumab use in psoriasis patients. Journal of the American Academy of Dermatology. 2011;65(3):546–51. 10.1016/j.jaad.2010.05.033 . 21514689

[19] Wolbink GJ, Vis M, Lems W, Voskuyl AE, de Groot E, Nurmohamed MT, et al Development of antiinfliximab antibodies and relationship to clinical response in patients with rheumatoid arthritis. Arthritis and rheumatism. 2006;54(3):711–5. 10.1002/art.21671 . 16508927

[20] Kosmac M, Avcin T, Toplak N, Simonini G, Cimaz R, Curin Serbec V. Exploring the binding sites of anti-infliximab antibodies in pediatric patients with rheumatic diseases treated with infliximab. Pediatric research. 2011;69(3):243–8. 10.1203/PDR.0b013e318208451d . 21131896

[21] van Schouwenburg PA, Rispens T, Wolbink GJ. Immunogenicity of anti-TNF biologic therapies for rheumatoid arthritis. Nature reviews Rheumatology. 2013;9(3):164–72. 10.1038/nrrheum.2013.4 . 23399692

[22] Douni E, Sfikakis PP, Haralambous S, Fernandes P, Kollias G. Attenuation of inflammatory polyarthritis in TNF transgenic mice by diacerein: comparative analysis with dexamethasone, methotrexate and anti-TNF protocols. Arthritis research & therapy. 2004;6(1):R65–R72. 10.1186/ar1028 . 14979939 PMC400419

[23] Bongartz T, Sutton AJ, Sweeting MJ, Buchan I, Matteson EL, Montori V. Anti-TNF antibody therapy in rheumatoid arthritis and the risk of serious infections and malignancies: systematic review and meta-analysis of rare harmful effects in randomized controlled trials. JAMA. 2006;295(19):2275–85. 10.1001/jama.295.19.2275 . 16705109

[24] Ali T, Kaitha S, Mahmood S, Ftesi A, Stone J, Bronze MS. Clinical use of anti-TNF therapy and increased risk of infections. Drug, healthcare and patient safety. 2013;5:79–99. 10.2147/DHPS.S28801 . 23569399 PMC3615849

[25] Pasparakis M, Alexopoulou L, Episkopou V, Kollias G. Immune and inflammatory responses in TNF alpha-deficient mice: a critical requirement for TNF alpha in the formation of primary B cell follicles, follicular dendritic cell networks and germinal centers, and in the maturation of the humoral immune response. The Journal of experimental medicine. 1996;184(4):1397–411. 10.1084/jem.184.4.1397 . 8879212 PMC2192824

[26] Chen IJ, Chuang CH, Hsieh YC, Lu YC, Lin WW, Huang CC, et al Selective antibody activation through protease-activated pro-antibodies that mask binding sites with inhibitory domains. Scientific reports. 2017;7(1):11587 10.1038/s41598-017-11886-7 . 28912497 PMC5599682

[27] Kalden JR, Schulze-Koops H. Immunogenicity and loss of response to TNF inhibitors: implications for rheumatoid arthritis treatment. Nature reviews Rheumatology. 2017;13(12):707–18. 10.1038/nrrheum.2017.187 . 29158574

[28] Bartelds GM, Krieckaert CL, Nurmohamed MT, van Schouwenburg PA, Lems WF, Twisk JW, et al Development of antidrug antibodies against adalimumab and association with disease activity and treatment failure during long-term follow-up. JAMA. 2011;305(14):1460–8. 10.1001/jama.2011.406 . 21486979

[29] Weinblatt ME, Maddison PJ, Bulpitt KJ, Hazleman BL, Urowitz MB, Sturrock RD, et al CAMPATH-1H, a humanized monoclonal antibody, in refractory rheumatoid arthritis. An intravenous dose-escalation study. Arthritis and rheumatism. 1995;38(11):1589–94. . 7488279 10.1002/art.1780381110

[30] Vidarsson G, Dekkers G, Rispens T. IgG subclasses and allotypes: from structure to effector functions. Frontiers in immunology. 2014;5:520 10.3389/fimmu.2014.00520 . 25368619 PMC4202688

[31] Ma X, Xu S. TNF inhibitor therapy for rheumatoid arthritis. Biomedical reports. 2013;1(2):177–84. 10.3892/br.2012.42 . 24648915 PMC3956207

[32] Yost J, Gudjonsson JE. The role of TNF inhibitors in psoriasis therapy: new implications for associated comorbidities. F1000 medicine reports. 2009;1 10.3410/M1-30 . 20948750 PMC2924720

[33] Kircik LH, Del Rosso JQ. Anti-TNF agents for the treatment of psoriasis. Journal of drugs in dermatology: JDD. 2009;8(6):546–59. . 19537380

[34] Levin AD, Wildenberg ME, van den Brink GR. Mechanism of Action of Anti-TNF Therapy in Inflammatory Bowel Disease. Journal of Crohn's & colitis. 2016;10(8):989–97. 10.1093/ecco-jcc/jjw053 . 26896086

[35] Choi KY, Swierczewska M, Lee S, Chen X. Protease-activated drug development. Theranostics. 2012;2(2):156–78. 10.7150/thno.4068 . 22400063 PMC3296471

[36] Fink K, Boratynski J. [The role of metalloproteinases in modification of extracellular matrix in invasive tumor growth, metastasis and angiogenesis]. Postepy higieny i medycyny doswiadczalnej. 2012;66:609–28. . 23001203 10.5604/17322693.1009705

[37] Wunder A, Tung CH, Muller-Ladner U, Weissleder R, Mahmood U. In vivo imaging of protease activity in arthritis: a novel approach for monitoring treatment response. Arthritis and rheumatism. 2004;50(8):2459–65. 10.1002/art.20379 . 15334458

[38] McKerrow JH, Bhargava V, Hansell E, Huling S, Kuwahara T, Matley M, et al A functional proteomics screen of proteases in colorectal carcinoma. Molecular medicine. 2000;6(5):450–60. . 10952024 PMC1949953

[39] Li W, Danilenko DM, Bunting S, Ganesan R, Sa S, Ferrando R, et al The serine protease marapsin is expressed in stratified squamous epithelia and is up-regulated in the hyperproliferative epidermis of psoriasis and regenerating wounds. The Journal of biological chemistry. 2009;284(1):218–28. 10.1074/jbc.M806267200 . 18948266

[40] Sanmarti R, Ruiz-Esquide V, Bastida C, Soy D. Tocilizumab in the treatment of adult rheumatoid arthritis. Immunotherapy. 2018;10(6):447–64. 10.2217/imt-2017-0173 . 29495891

[41] Blanco FJ, Moricke R, Dokoupilova E, Codding C, Neal J, Andersson M, et al Secukinumab in Active Rheumatoid Arthritis: A Phase III Randomized, Double-Blind, Active Comparator- and Placebo-Controlled Study. Arthritis & rheumatology. 2017;69(6):1144–53. 10.1002/art.40070 . 28217871

[42] Campbell L, Chen C, Bhagat SS, Parker RA, Ostor AJ. Risk of adverse events including serious infections in rheumatoid arthritis patients treated with tocilizumab: a systematic literature review and meta-analysis of randomized controlled trials. Rheumatology. 2011;50(3):552–62. 10.1093/rheumatology/keq343 . 21078627

[43] Vallabhaneni S, Chiller TM. Fungal Infections and New Biologic Therapies. Current rheumatology reports. 2016;18(5):29 10.1007/s11926-016-0572-1 . 27032792

[44] Zhu L, Xie J, Swierczewska M, Zhang F, Quan Q, Ma Y, et al Real-time video imaging of protease expression in vivo. Theranostics. 2011;1:18–27. . 21461134 10.7150/thno/v01p0018 PMC3068198

[45] Chen WC, Tseng CK, Chen BH, Lin CK, Lee JC. Grape Seed Extract Attenuates Hepatitis C Virus Replication and Virus-Induced Inflammation. Frontiers in pharmacology. 2016;7:490 10.3389/fphar.2016.00490 . 28066241 PMC5174132

[46] Hayward MD, Jones BK, Saparov A, Hain HS, Trillat AC, Bunzel MM, et al An extensive phenotypic characterization of the hTNFalpha transgenic mice. BMC physiology. 2007;7:13 10.1186/1472-6793-7-13 . 18070349 PMC2222242

[47] Keffer J, Probert L, Cazlaris H, Georgopoulos S, Kaslaris E, Kioussis D, et al Transgenic mice expressing human tumour necrosis factor: a predictive genetic model of arthritis. The EMBO journal. 1991;10(13):4025–31. . 1721867 10.1002/j.1460-2075.1991.tb04978.x PMC453150

[48] Shealy DJ, Wooley PH, Emmell E, Volk A, Rosenberg A, Treacy G, et al Anti-TNF-alpha antibody allows healing of joint damage in polyarthritic transgenic mice. Arthritis research. 2002;4(5):R7 10.1186/ar430 . 12223110 PMC125301

