# Supplementary figures and images for: Specific activation of pro-Infliximab enhances selectivity and safety of rheumatoid arthritis therapy
Source: PLoS Biol. 2019 Jun 13;17(6):e3000286. doi: 10.1371/journal.pbio.3000286 (PMC6563948; doi:10.1371/journal.pbio.3000286)

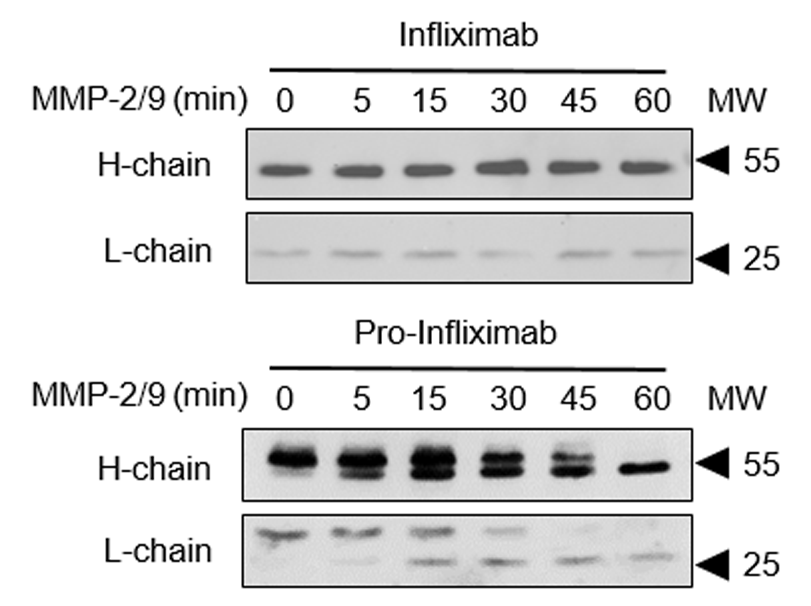

Supplement: S1 Fig — To investigate whether the Ab lock can be efficiently removed from pro-Infliximab and restore the antigen-binding ability of pro-Infliximab after MMP-2/9 cleavage, we incubated pro-Infliximab and Infliximab with MMP-2/9 for different time periods and evaluated the MW profile of Ab fragments by western blot. The results showed that the heavy-chain (58 kDa) and light-chain (29.4 kDa) molecular weight of pro-Infliximab was converted into a profile similar to the control Infliximab (55 kDa for heavy chain and 25.6 kDa for light chain) after treatment with MMP-2/9 within 60 min, demonstrating that MMP-2/9 could completely remove the Ab lock from pro-Infliximab. Ab, antibody; MMP, matrix metalloproteinase; MW, molecular weight. (TIF) [file pbio.3000286.s001.tif]

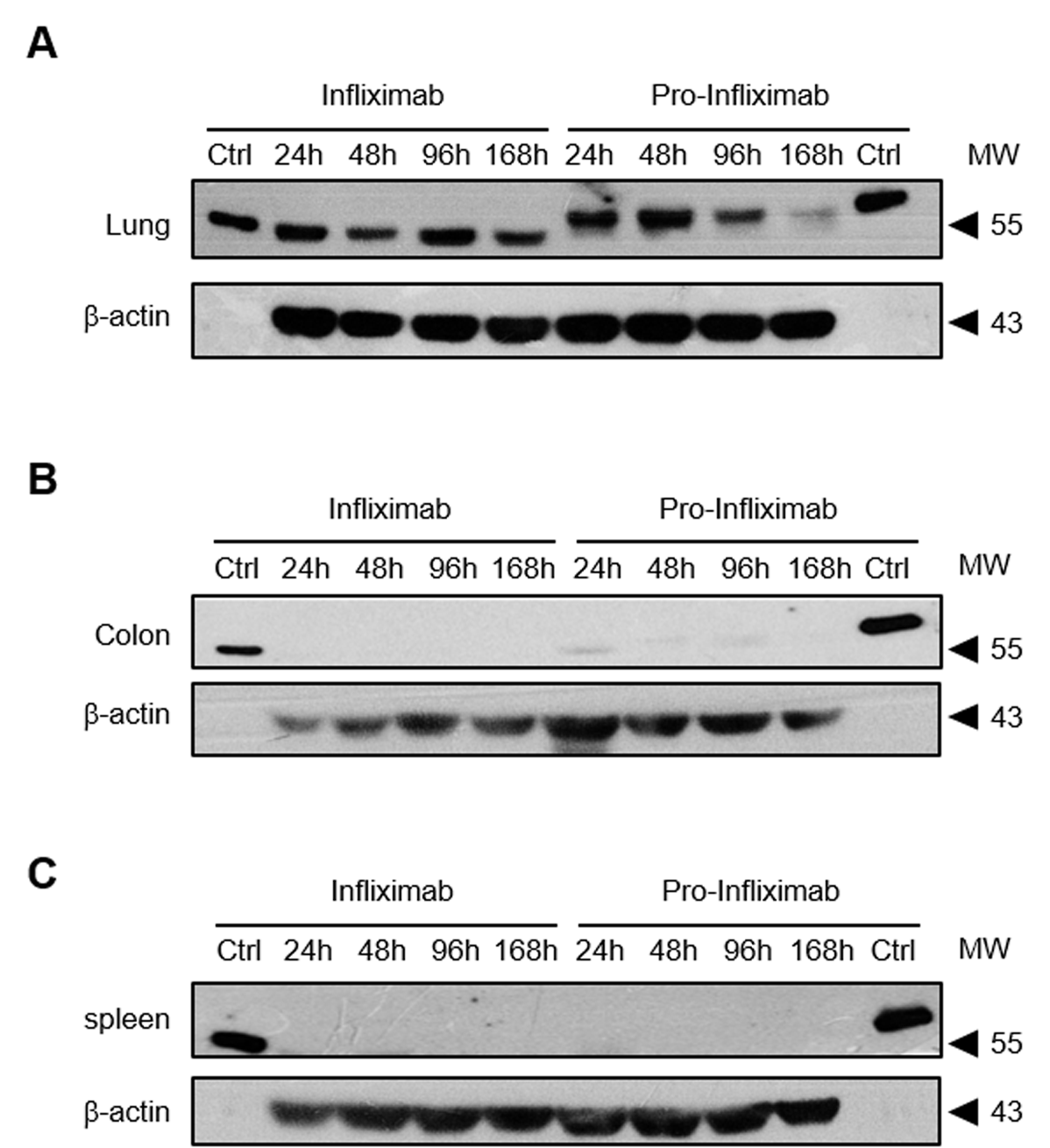

Supplement: S3 Fig — hTNFα-transgenic 1006 mice were intraperitoneally injected with 50 μg Infliximab or pro-Infliximab. After 24, 48, 96, and 168 h, the (A) lung, (B) colon, and (C) spleen tissue were collected using HRP-conjugated anti-human IgG Fc Ab for detecting the level of active and inactive pro-Infliximab by western blot. The β-actin as internal control. Ab, antibody; Fc, fragment crystallizable; HRP, horseradish peroxidase; IgG, immunoglobulin; TNFα, tumor necrosis factor α. (TIF) [file pbio.3000286.s003.tif]
