# Supplementary material for: Specific activation of pro-Infliximab enhances selectivity and safety of rheumatoid arthritis therapy
Source: PLoS Biol. 2019 Jun 13;17(6):e3000286. doi: 10.1371/journal.pbio.3000286 (PMC6563948; doi:10.1371/journal.pbio.3000286)
Supplement: S1 Text — (DOCX) [file pbio.3000286.s007.docx]

Specific activation of pro-Infliximab enhances selectivity and safety of rheumatoid arthritis therapy

*Yun-Chi Lu, Chih-Hung Chuang, Kuo-Hsiang Chuang, I-Ju Chen, Bo-Cheng Huang, Wen-Han Lee, Hsin-Ell Wang, Jia-Je Li, Yi-An Cheng, Kai-Wen Cheng, Jaw-Yuan Wang, Yuan-Chin Hsieh, Wen-Wei Lin, Tian-Lu Cheng*

**Supplementary Information**

**Supplementary Methods**

*Pro-anti-IL6 receptor Ab construction, expression, and purification*

The complementary DNA coding for the heavy and light chains of anti-IL6 receptor Ab were cloned through assembly PCR. The hinge-encoding sequences, GGGGS linker, and MMP-2/9 substrate-encoding sequences (GPLGVR)[[1](#_ENREF_1)] were introduced upstream of the light chain and heavy chain to generate pro- anti-IL6 receptor Ab. The anti-IL6 receptor Ab or pro-anti-IL6 receptor Ab production were through the Expi293 Expression System (Thermo Fisher Scientific) and they were purified using Protein A-Sepharose (GE Healthcare, Milwaukee, WI, USA).

*Comparison of the binding ability of the pro- anti-IL6 receptor Ab with or without MMP-2/9 treatment*

To determine the binding kinetics (EC_50_) of pro-anti-IL6 receptor Ab and anti-IL6 receptor Ab. The recombinant IL-6 receptor (Abcam, Cambridge, MA) was coated onto 96-well plates and blocked with 5% skim milk. The anti-IL6 receptor Ab or pro-anti-IL6 receptor Ab were incubated with or without 20 μg/mL of MMP-2/9 in DMEM/0.05% BSA (pH: 7.4) for 1 h at 37°C before the reaction was terminated by BCS. All the samples were added onto the plates at the given concentrations, and ELISA was performed as described previously.

*Immunogenicity of human immune cells to Infliximab, pro-Infliximab and MMP-2/9 substrate linker.*

To prepare monocyte-derived dendritic cells (DCs), peripheral blood mononuclear cells (PBMCs) from healthy donor blood isolated by Ficoll-Paque and monocytes isolated using Miltenyi Pan Monocyte Isolation Kits (Miltenyi Biotech) and LS columns (Miltenyi Biotech) according to manufacturer's instructions. Monocytes were resuspended in RPMI1640 supplemented with 10% FCS, 2 mM L-glutamine, 100 units/ml penicillin/streptomycin, 2.5 μg/ml Fungizone and 500 units/ml recombinant human IL-4 (Invitrogen) and 500 units/ml recombinant human granulocyte-macrophage colony-stimulating factor (R&D systems) (DC culture medium), and then seeded in 24-well plate at a density of 4 × 10^5^ cells/well. On day 3, renew the DC culture medium. On day 6, 20 ng/ml recombinant human TNF-α (Sigma) and 10 ng/ml IL-1β (R&D systems) were added to the cells to activate DCs for 24 h. On day 7, the harvested DCs were counted and then incubated with final concentration of 50 μg/ml mitomycin C for 30 min at 37°C at a density of 1 × 10^6^ cells/ml, then washed extensively. Autologous CD4^+^ T cells were isolated on Day 7 by negative selection from frozen PBMCs using CD4^+^ T Cell Isolation Kit II (Miltenyi Biotech) and LS columns according to the manufacturer's instructions. After counting, 2 × 10^5^ CD4^+^ T Cells were added to 2 × 10^4^ mitomycin C-treated DCs and incubated with pro-Infliximab, Infliximab or MMP-2/9 substrate linker (MMP-2/9 SL) at concentration of 350 nM in 96-well round bottom plates. Controls included dendritic cells plus CD4^+^ T cells alone and with concentration of 10 μg/ml phytohemagglutinin (PHA). Cells were cultured at 37°C in 5% carbon dioxide for 5 days. Proliferation was assessed by ATPlite Luminescence Assay kit (Perkin Elmer) according to manufacturer's instructions. Counts per minute (CPM) for each well were determined by multimode plate reader (Perkin Elmer).**Supplemental References**

1. Zhu L, Xie J, Swierczewska M, Zhang F, Quan Q, Ma Y, et al. Real-time video imaging of protease expression in vivo. Theranostics. 2011;1:18-27. PubMed PMID: 21461134; PubMed Central PMCID: PMC3068198.
